# A TEX_86_ surface sediment database and extended Bayesian calibration

**DOI:** 10.1038/sdata.2015.29

**Published:** 2015-06-23

**Authors:** Jessica E Tierney, Martin P Tingley

**Affiliations:** 1 Woods Hole Oceanographic Institution, 266 Woods Hole Road, Woods Hole, MA 02543, USA; 2 The University of Arizona, Department of Geosciences, 1040 E 4th St Tucson, AZ 85721, USA; 3 Pennsylvania State University, Departments of Statistics and Meteorology, 510 Walker Building, University Park, PA 16802, USA

**Keywords:** Palaeoceanography, Biogeochemistry, Climate change, Palaeoclimate

## Abstract

Quantitative estimates of past temperature changes are a cornerstone of paleoclimatology. For a number of marine sediment-based proxies, the accuracy and precision of past temperature reconstructions depends on a spatial calibration of modern surface sediment measurements to overlying water temperatures. Here, we present a database of 1095 surface sediment measurements of TEX_86_, a temperature proxy based on the relative cyclization of marine archaeal glycerol dialkyl glycerol tetraether (GDGT) lipids. The dataset is archived in a machine-readable format with geospatial information, fractional abundances of lipids (if available), and metadata. We use this new database to update surface and subsurface temperature calibration models for TEX_86_ and demonstrate the applicability of the TEX_86_ proxy to past temperature prediction. The TEX_86_ database confirms that surface sediment GDGT distribution has a strong relationship to temperature, which accounts for over 70% of the variance in the data. Future efforts, made possible by the data presented here, will seek to identify variables with secondary relationships to GDGT distributions, such as archaeal community composition.

## Background & Summary

The reconstruction of past changes in ocean temperatures allows us to understand the behavior of the Earth’s climate system, including internal and external drivers of oceanic variability, climate sensitivity, and ocean-atmosphere interactions. A number of techniques are available to infer past temperatures from ocean sediment archives, some employing the inorganic chemical composition of calcified fossils^1,2^, and others the distribution of fossil lipids, or ‘biomarkers’, produced by specific organisms^3,4^. The latter category includes the TEX_86_ (TetraEther indeX of 86 carbons) proxy, based on the relative cyclization of isoprenoidal glycerol dialkyl glycerol tetraethers (GDGTs) produced by marine archaea. GDGTs are cell membrane lipids, and archaea alter the composition of these lipids in response to environmental temperature in order to optimize membrane packing and fluidity^5–7^. Mesocosm experiments demonstrate that marine archaea produce relatively more lipids with a greater number of rings at higher temperatures^8,9^.

**Figure 1:**
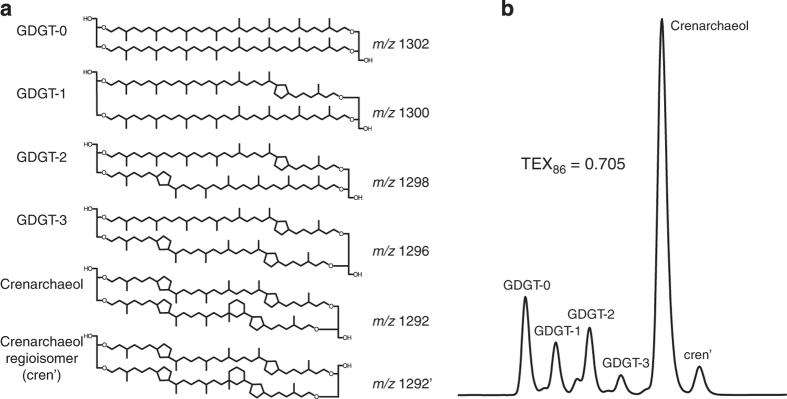
Molecular structures and HPLC detection of GDGTs. (**a**) Structures of the isoprenoidal GDGTs that comprise the TEX_86_ proxy, along with Crenarchaeol, a diagnostic lipid for Thaumarchaeota^10^. (**b**) A typical HPLC-MS trace of the isoprenoidal GDGTs and corresponding TEX_86_ value.

TEX_86_ is an index designed to quantify the relative degree of cyclization^4^. It is defined as:

(1)TEX86=GDGT−2+GDGT−3+cren′GDGT−1+GDGT−2+GDGT−3+cren′

where GDGTs 1–3 are compounds containing 1–3 cyclopentyl moieties, respectively, and *cren'* denotes the regioisomer of crenarchaeol, a characteristic lipid for Thaumarchaeota^10^. By definition, values of the TEX_86_ index span 0–1. Fig. 1a shows the structures of these compounds. GDGTs are analyzed via High-Performance Liquid Chromatography-Mass Spectrometry (HPLC-MS); Fig. 1b shows their typical appearance in a HPLC-MS chromatogram.

Pelagic, nitrifying Thaumarchaeota are believed to be the primary^10,11^, but likely not exclusive^12^ producers of GDGTs in the marine environment. These organisms typically inhabit the upper water column, but may reside anywhere in the epi- or meso-pelagic zone^13–15^. The strong empirical relationship between TEX_86_ and sea-surface temperatures (SSTs)^4,16–19^ has led to the widespread use of TEX_86_ to reconstruct past SSTs on both recent^20^ and ancient timescales^21^. However, in environments with steep thermoclines and nutriclines, Thaumarchaeota may reside deeper in the water column (e.g., 50–200 meters depth) and record subsurface temperature variability^11,19,22–25^. Therefore, TEX_86_ may be used to reconstruct either SST or subsurface temperatures, depending on the oceanographic conditions.

Calibration of the TEX_86_ index to temperatures relies on a collection of modern surface sediments, for which overlying water temperatures are known from historical observations^4,16–18^. Modern surface data are continuously published in disparate journals, making aggregation of the data for calibration purposes difficult. Here, we present a database of 1095 surface sediment TEX_86_ measurements, which may be used to calibrate the TEX_86_ proxy and investigate relationships between TEX_86_ and other environmental variables. We also present updated versions of the BAYSPAR (Bayesian, Spatially-Varying Regression)^18^ calibration for TEX_86_ based on this new data collection, including both surface temperature (SST) and subsurface temperature (Sub-T) models.

## Methods

### Data aggregation

**Figure 2:**
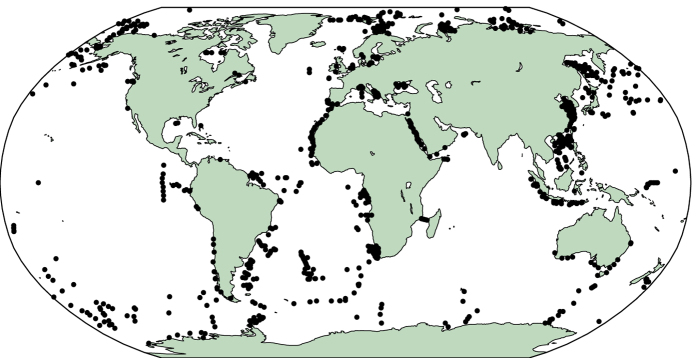
The distribution of sedimentary core top data in the global TEX_86_ database.

TEX_86_ data (*n*=1095) were collated from the literature and from direct contact with individual researchers (Fig. 2). Our collection includes data represented in previous global calibration efforts^16–18^, data published as part of regional surface sediment studies^24,26–42^, surface sediment data produced as part of a sedimentary TEX_86_ timeseries^43–48^, and previously unpublished data. The TEX_86_ measurements in this database were reported by the original authors and contributors to be modern or at the least, late Holocene in age, and therefore generally representative of present-day temperatures. All TEX_86_ data entries are accompanied by geospatial information. In some cases, authors archived the relative abundances of individual compounds. We compiled this information when available (see Data Records below).

### Analytical determination of TEX_86_

Although the data in this collection derive from multiple publications and laboratories, TEX_86_ values were determined using the same HPLC-MS analysis method^49^. Briefly, extracts of sediment material containing GDGTs were dissolved in a mixture of hexane and isopropanol, injected into a HPLC, then separated on a Prevail Cyano column using a gradient spanning hexane:isopropanol (99:1) to hexane:isopropanol (98.2:1.8). The solvent stream is then sent to a mass spectrometer operated in single-ion monitoring (SIM) mode, scanning only target compound mass-to-charge ratios. The type of mass spectrometer (e.g., single quadrupole, ion trap) may be different between laboratories, but previous research has shown that there is no bias in TEX_86_ associated with different types of mass spectrometers^50^. TEX_86_ is calculated from integrating peak areas of the target compounds. Within a single laboratory, analytical error is typically 0.004 TEX_86_ units or better^50,51^, or about 0.3 °C when calibrated. Interlaboratory uncertainties are nearly an order-of-magnitude larger (0.03 TEX_86_ units^50,51^), equivalent to about 2–3 °C.

### BAYSPAR calibration model

We have previously developed a Bayesian, spatially-varying regression (BAYSPAR) model^18^ for the calibration of TEX_86_. The adoption of this model was motivated by observations that the TEX_86_ response to temperature varies across different oceanic basins and environments^26,35^, the existence of strong spatial trends in the residuals of previous calibration models^18,19^, and the need to fully propagate uncertainties into resulting temperature predictions.

BAYSPAR assumes the regression parameters are constant within 20° by 20° latitude-longitude grid boxes, but imposes a spatial model on the intercepts (the vector ***α***) and slopes (***β***) that forces nearby grid boxes to feature similar parameter values, with the degree of similarity controlled by a data-informed spatial decorrelation length scale. This hierarchical approach produces a calibration that is a data-determined compromise between a globally constant calibration and a set of independent local calibrations^52,53^. The calibration model is specified via the following set of equations:

(2)P=Mα+MCβ+Ɛ
,

(3)Ɛ
∼N(0,τ2I),

(4)α∼N(µα1,σα2R(ν,ϕ)),

(5)β∼N[0,∞)(µβ1,σβ2R(ν,ϕ)),

The vector **P** consists of all core-top TEX_86_ observations; **C** is a diagonal matrix containing all temperature observations; and **M** is a selection matrix of zeros and ones, with each row containing a single one, such that corresponding entries of the vectors MC***β*** and ***P*** are at the same location in space. ***α*** and ***β*** are, respectively, vectors of spatially varying intercept and slope terms; along with the error variance, *τ* ^2^ (I denotes the identity matrix), they are the parameters of primary interest in calibrating the TEX_86_–temperature relationship. Spatial dependence arises from the specification of both ***α*** and ***β*** as stationary and isotropic Gaussian processes in space, defined on the centroids of 20° by 20° grid boxes, and with constant means given by *μ*_*α*_ and *μ*_*β*_, respectively. $N_{[0,\infty )}$ indicates a truncated normal, defined on the positive half of the real line, reflecting the *a priori* assumption of a positive relationship between TEX_86_ and temperatures. Finally, R denotes the Matérn correlation function^53^, defined by a smoothness parameter *ν*, which we set to 3/2, and an inverse spatial range parameter, ϕ, that measures the strength of the spatial dependence. To provide mathematical closure, priors are required for all scalar parameters of the calibration model. With the exception of ϕ, which can be challenging to estimate^54–56^, we use proper but weakly informative priors.

Prediction of temperature conditional on an observed TEX_86_ value proceeds by a second application of Bayes rule to invert Equation 2 for temperature in terms of TEX_86_. A prior distribution on the temperature is also required, and, to propagate uncertainty, we integrate over the posterior distributions of the calibration parameters. In practice, this is achieved by repeatedly sampling from the posterior distributions of the calibration parameters, and then drawing from the posterior predictive distribution of temperatures conditional on the TEX_86_ observation, the current draw of the calibration parameters, and the prior on past temperature.

**Figure 3:**
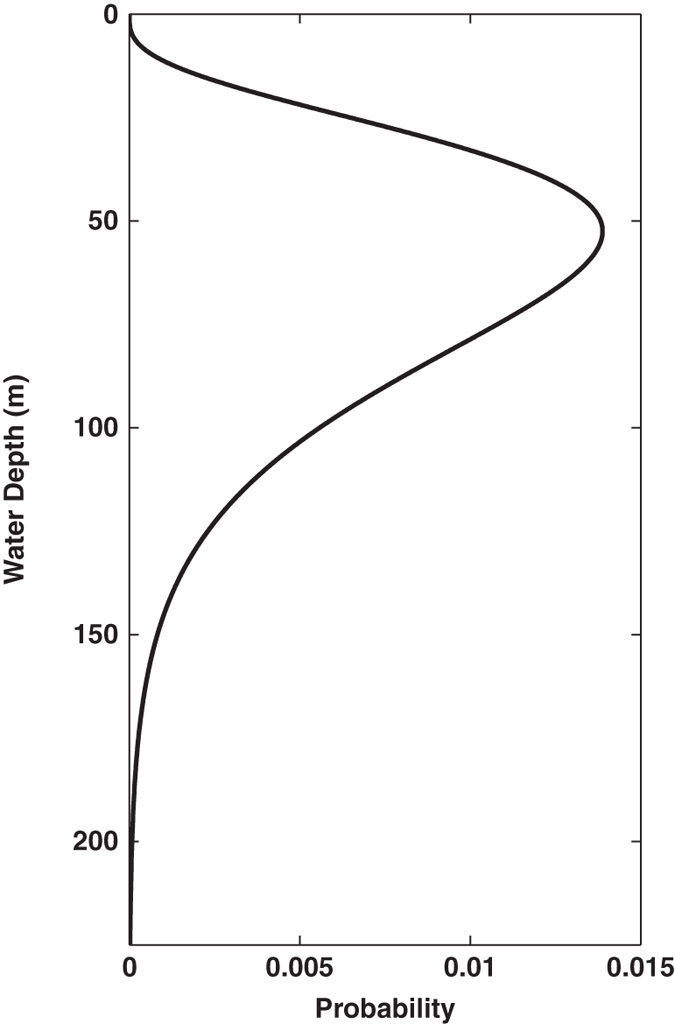
Gamma function probability distribution that represents the averaging scheme for the subsurface temperature calibration. The gamma distribution parameters are a=4.5 and b=15.

Under certain oceanographic conditions, TEX_86_ may be recording subsurface, rather than surface, temperature variability^11,19,22–24^. Several subsurface calibrations have been proposed in the past^16,23,25^. We therefore present separate calibrations of the BAYSPAR model using both modern SST climatologies, and a modern climatology of sub-surface temperatures (Sub-T). The formalism is the same in each case, except that, for the Sub-T calibration, the target temperatures are set as weighted averages of the 0–200 meters water depth, with weights given by the gamma probability density function (Fig. 3). We chose this weighting function to approximate evidence from water column studies that GDGT production occurs predominantly between 0–200 meters but likely reaches peak abundance in the shallow subsurface^24,57,58^. Initial experiments using a simple average between 0–200 meters resulted in poor fit, especially in shallow regions of the global ocean (not shown). In keeping with previous findings that TEX_86_ has a weak relationship to temperatures in the high latitudes of the Arctic ocean^18,35^ we exclude data north of 70° N in both calibration models.

## Data Records

The TEX_86_ surface sediment database is archived at the National Oceanic and Atmospheric Administration's National Climatic Data Center for Paleoclimatology: http://www.ncdc.noaa.gov/paleo/study/18615 in machine-readable ASCII format (http://www.ncdc.noaa.gov/data-access/paleoclimatology-data/contributing). The database is also archived on Figshare (Data Citation 1). Each data entry includes the following information:

1. Geospatial information, including latitude, longitude and (if available) recorded water depth at the collection site.
2. Sediment core information, including the name of the core, type of core (e.g., gravity, piston), and depth at which the TEX_86_ sample was taken.
3. TEX_86_ value and (if available) fractional abundances of the six main isoprenoidal GDGTs.
4. Overlying sea-surface temperatures and gamma-averaged (Fig. 3) subsurface temperatures derived from the 1°×1° World Ocean Atlas 2009 product^59^ (https://www.nodc.noaa.gov/OC5/WOA09/pr_woa09.html) and sea-surface temperatures from the 0.25°×0.25° NOAA daily Optimum Interpolation Sea Surface Temperature (OISST) 1981–present climatology based on Advanced Very-High Resolution Radiometer (AVHRR) measurements^60^ (http://www.ncdc.noaa.gov/oisst).
5. Name and DOI of the associated reference, if available.

The database includes all available sedimentary TEX_86_ data as of January 2015. This version of the database and the accompanying calibrations is designed as version 1.0. The authors will update the database, and the BAYSPAR calibrations, yearly with newly published sediment core top data; previous versions of the database and calibrations will be archived at the NCDC for posterity.

## Technical Validation

**Figure 4:**
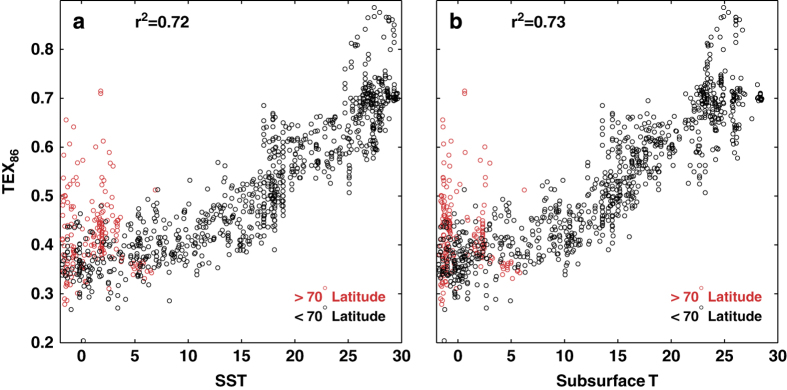
Scatterplots of the TEX_86_ database versus a. sea-surface temperature (SST), and b. subsurface (Sub-T; 0–200 meters, gamma averaged) temperature. Red dots denote data located above 70°N latitude; black dots denote all other data.

The new TEX_86_ data compilation shows a clear relationship with both SST and Sub-T (subsurface temperatures), which respectively account for 72 and 73% of the variance in the TEX_86_ data (Fig. 4). The relationship is not straightforwardly linear due to regional differences in the TEX_86_-temperature slope. In particular, and in agreement with previous findings^18,35^, the TEX_86_-temperature relationship features a lower slope at higher latitudes, and there is more scatter about the regression relationship in the Arctic region (Fig. 4). The reasons for the poor relationship between TEX_86_ and temperatures in the Arctic remain unclear. In some locations it may reflect interference from terrestrial or sedimentary methanogenic/methanotrophic sources of GDGTs^35^ but could also plausibly indicate the presence of different pelagic archaeal producers. Whatever the case, the scatter in the data and the subsequent collapse in predictability^18^ justify their current exclusion from global calibration models.

**Figure 5:**
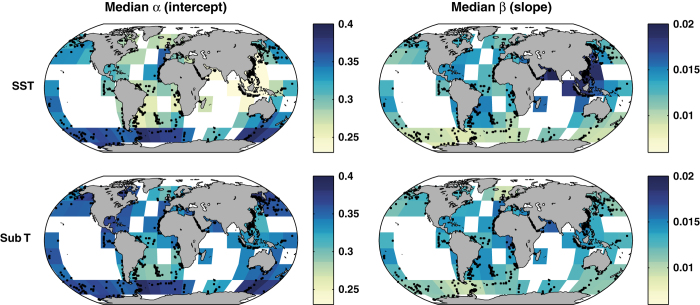
Spatially-varying median *α* (intercept) and *β* (slope) values calculated by BAYSPAR, for the SST model (top) and subsurface temperature (Sub-T) model (bottom). Small black dots denote the locations of the surface sediment TEX_86_ data.

In agreement with our previous work^18^, both the SST and Sub-T (subsurface temperature) BAYSPAR calibrations show spatial variation in the ***α*** (intercept) and ***β*** (slope) parameters that reflect the regional differences in the TEX_86_ response to temperature variations (Fig. 5). Globally, for the SST (Sub-T) model, ***β*** varies by 30% (22%) and ***α*** varies by 22% (10%). The relatively smaller variance of the parameters in the Sub-T model, particularly in the case of ***α***, may indicate a slightly less globally-variable TEX_86_ response when calibrating to a deeper water temperature.

**Figure 6:**
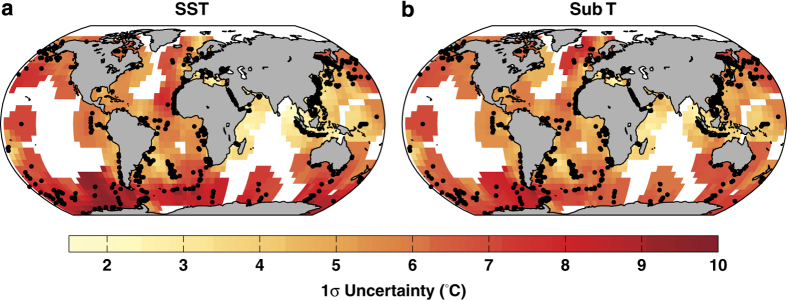
Spatially-varying 1*σ* uncertainties (**a**) and residuals (**b**) for the SST and Sub-T BAYSPAR models. Black dots denote the locations of the surface sediment TEX_86_ data.

Calibration uncertainties vary spatially as a function of data availability, and as a function of ***β***, with lower ***β*** values associated with higher uncertainties (Fig. 6). For the SST model, calibration uncertainties vary between 1.2–10 °C with a median of 5 °C; for the Sub-T model, they vary between 1.4–9 °C with a median of 5 °C. Unlike the existing least squares calibrations^16,17,61^, and in agreement with our previous calibrations^18^, we do not detect any significant trends in the residuals as a function of latitude (for the SST model, *ρ*=−0.07, *P*=0.11, while for the sub T model, *ρ*=−0.05, *P*=0.27, where ρ is the Spearman correlation).

**Figure 7:**
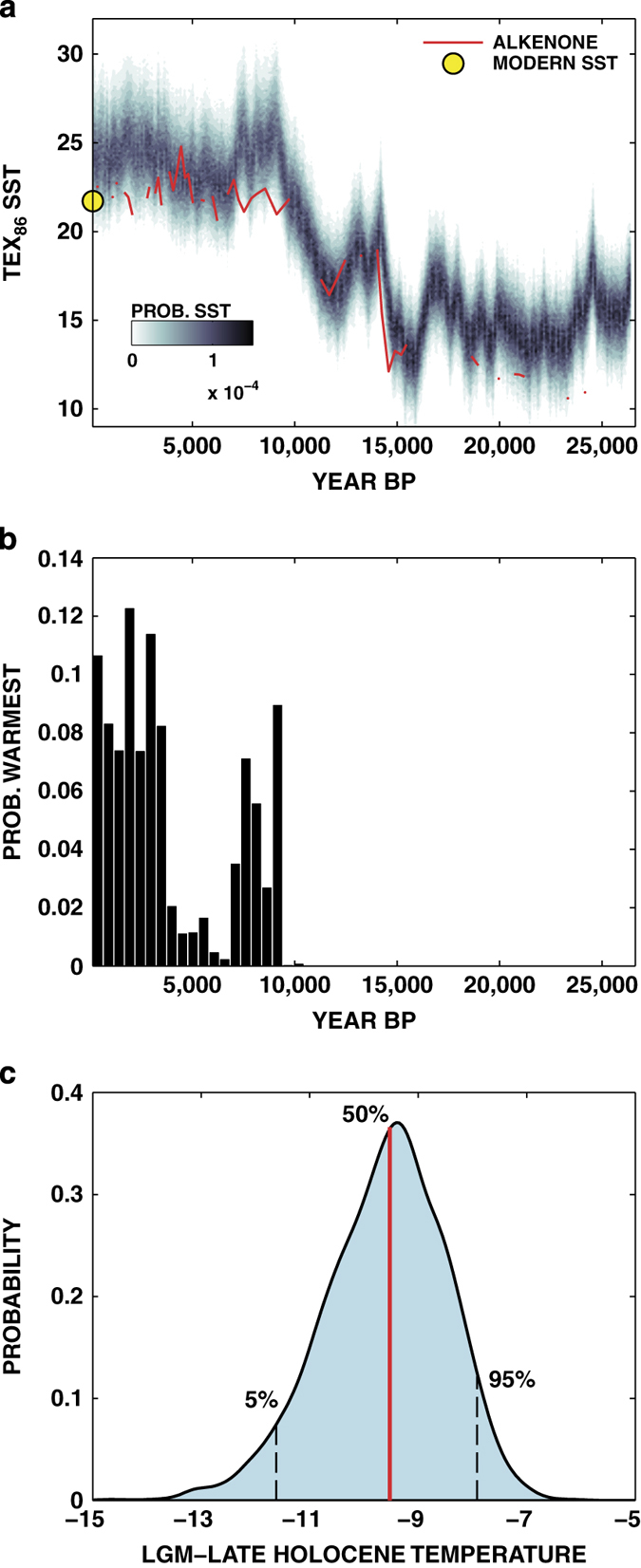
An example application of the TEX_86_ BAYSPAR model. (**a**) Posterior SST probability densities, derived from the application of the SST calibration to TEX_86_ data from the eastern Mediterranean^44^. Alkenone-based SST estimates (in red) are shown for comparison, and the yellow dot denotes WOA09 modern mean annual SST^59^. (**b**) Probability that each time point featured the warmest conditions over the time span of data, binned by 500 year intervals. (**c**) Probability density of LGM-Late Holocene temperatures, showing 5th, 50th, and 95th percentiles.

We provide an example application of the new BAYSPAR calibration, based on the updated TEX_86_ core top dataset, to demonstrate applicability and usage (Fig. 7). In this case, we apply the SST calibration to predict SSTs for the past 25,000 years at a site in the eastern Mediterranean^44^. We find that the predicted temperatures are in reasonable agreement with independent alkenone-based SST estimates down core (Fig. 7a), indicating that the use of an SST model at this site is appropriate. One advantage of our Bayesian approach is that predictions take the form of posterior probability distributions as opposed to single time series with error bars (Fig. 7a). Probabilistic reconstructions of this form permit for a statistically rigorous assessment of a much broader array of scientific issues^62–65^. For example, we can estimate the probability that the late Holocene time period (0–4 ka) was the warmest period of the past 25,000 years by identifying the warmest time point in each ensemble member. We find that intervals throughout the Holocene feature non-negligible probabilities of experiencing the warmest conditions, such that we cannot conclude at any reasonable level of significance that the late Holocene was the warmest period (Fig. 7b). In addition, we can estimate the magnitude of the LGM-Holocene temperature difference at this location that fully accounts for the uncertainties in the proxy estimates (Fig. 7c). The posterior median for LGM cooling is −9.5 °C, with a 90% uncertainty interval of (−11.6, −7.9) °C.

The performance of our new BAYSPAR calibrations and their application demonstrate the general ability of the new TEX_86_ database to provide predictions of past changes in both surface and subsurface temperatures. The choice of whether to calibrate to surface or sub-surface temperatures is ultimately up to the user, although we recommend that it be informed not only by the target variable that the user seeks to predict but also an understanding of the oceanography of the location from which the data derive. As previous investigations have shown^22,28^, a Sub-T calibration is likely the most suitable choice for regions with steep thermoclines and nutriclines, such as upwelling zones. The database may also foster future investigations into secondary influences on the distribution of isoprenoidal GDGTs in marine sediments, such as lipid contributions from different archaeal communities^12,66,67^.

## Usage Notes

Updated Matlab code that enables users to apply the latest BAYSPAR calibrations is available for download at Figshare: http://dx.doi.org/10.6084/m9.figshare.1348830. The BAYSPAR calibration may also be used online at http://www.whoi.edu/bayspar.

## Additional Information

**How to cite this article:** Tierney, J. E. & Tingley, M. P. A TEX_86_ surface sediment database and extended Bayesian calibration. *Sci. Data* 2:150029 doi: 10.1038/sdata.2015.29 (2015).

## Supplementary Material
